# Inducing Chirality in n‑Type Conjugated Polymers by Chiral Solvents for the Development of Spin Transistors

**DOI:** 10.1021/acsami.6c02690

**Published:** 2026-04-09

**Authors:** Jeongwoo Beak, Yina Moon, Justin J. O’Neil, Dongseong Yang, Brian P. Bloom, Minwoo Lee, Geon Chang Song, Yunseul Kim, David H. Waldeck, Dong-Yu Kim

**Affiliations:** † School of Materials Science and Engineering (SMSE), Research Institute for Solar and Sustainable Energies (RISE), Gwangju Institute of Science and Technology (GIST), Gwangju 61005, Republic of Korea; ‡ Department of Chemistry, Purdue University, 560 Oval Drive, West Lafayette, Indiana 47907, United States; § Department of Chemistry, 6614 University of Pittsburgh, 219 Parkman Avenue, Pittsburgh, Pennsylvania 15260, United States; ∥ User Convenience Technology R&D Department, Korea Institute of Industrial Technology (KITECH), Ansan-si 15588, Republic of Korea; ⊥ Division of Advanced Materials, Korea Research Institute of Chemical Technology (KRICT), 141 Gajeong-ro, Yuseong-gu, Daejeon 34114, Republic of Korea; # Andlinger Center for Energy and the Environment, Princeton University, Princeton, New Jersey 08540, United States

**Keywords:** chiral-induced spin selectivity (CISS), conjugated polymer, chiral solvent, spin polarization, spin transistor

## Abstract

In this work, chiral fibrils are shown to manifest in P­(NDI2OD-T2) polymers through the addition of chiral solvents and an off-center spin coating method. The aligned polymeric chains form without a residual chiral solvent in the films, and circular dichroism spectroscopy reveals successful chiral imprinting. Spectroscopic measurements point to rotation of the conjugated polymer backbone, along with modified lamellar stacking, which promotes enhanced crystallinity along the backbone in a chiral fashion. Magneto field-effect transistors (mFETs), composed of chiral conjugated polymers, showed a strong dependence on the orientation of the externally applied magnetic field, leading to a robust asymmetric response in spin polarization current and charge carrier mobility that was sensitive to the handedness of the polymer. These findings are rationalized within the context of the chiral-induced spin-selectivity (CISS) effect. Collectively, this study establishes design strategies for organizing achiral conjugated polymers into chiral assemblies and provides a pathway for their implementation in spintronic device applications.

## Graphical abstract

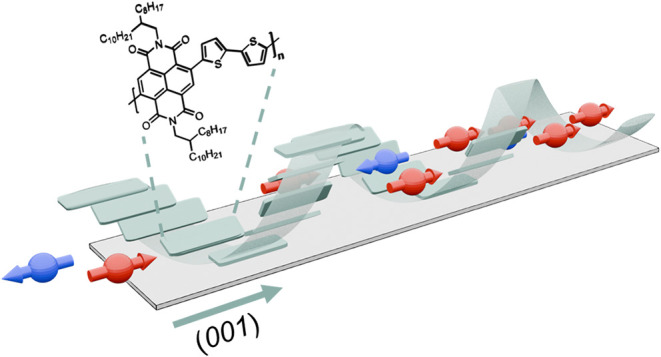

## 1 Introduction

Chiral conjugated polymers have emerged as promising candidates for next-generation technologies, including photodetectors, cryptographic systems, and spintronic devices.^1–9^ In addition, recent studies have shown that this interesting class of materials can also preferentially transmit electrons with one spin orientation over that of the other,^10–12^ a phenomenon referred to as the chiral-induced spin selectivity (CISS) effect.^13^ Because of the structural flexibility of chiral conjugated polymers, these materials are useful for investigating how the chiroptical properties, molecular length, and alignment of the polymer affect spin-filtering. Herein, we explore the CISS properties of chiral conjugated polymers through a magneto field-effect transistor approach.^14,15^

The most commonly employed method for inducing chirality in achiral conjugated polymers is to blend the materials with chiral small molecules,^16–23^ however, in most instances the chiral molecules are not removed after post processing, and this can make it difficult to deconvolve the chiroptical response from the individual components.^24–26^ Furthermore, cocrystallization strategies between the conjugated polymer and chiral small molecules often lead to reduced charge transport properties compared to those of the pristine achiral conjugated polymer. For these reasons, it is necessary to develop strategies for chiral induction in achiral conjugated polymers, and their respective polymer films,^27–29^ free from residual chiral molecules, while preserving or even enhancing the charge transport properties.

In this work, chirality induction was achieved in the achiral n-type conjugated polymer P­(NDI2OD-T2), using the chiral solvent N-benzyl-1-phenylethylamine (BPA) in tandem with an off-center spin coating method to uniaxially align the resulting fibrillar structures of the polymer.^30^ To the best of our knowledge, this is the first reported instance of BPA being used as a chiral solvent for inducing chirality in conjugated polymers. Because of the volatility of BPA, the chiral solvent could be removed after postfilm formation, leading to pristine aligned chiral polymer fibrils. Systematic structural and morphological analyses revealed that the chiral solvent induced a pronounced structural rearrangement of the conjugated polymer. Integration of the optimized chiral conjugated polymers into magneto field-effect transistors (mFET) showed a spin dependence of the current and the mobility with the orientation of an applied external magnetic field (North vs South) that depended sensitively on the enantiomeric form. Notably, the highest asymmetry occurred near the threshold voltage and decreased at higher operating voltages, suggesting a rich relationship between the charge transport dynamics and spin selectivity. This study demonstrates a scalable, residue-free strategy for chirality induction to fabricate aligned chiral-induced conjugated polymer films with spin transport characteristics.

## 2 Results and Discussion

### 2.1 Formation of Aligned Chiral Conjugated Polymers

Chirality induction in P­(NDI2OD-T2) was achieved through blending with (*S*)- and (*R*)-(±)-*N*-benzyl-1-phenylethylamine (BPA) in bromobenzene (BrB) followed by spin coating using an off-center spin coating method (Figure 1a,b). Films were fabricated using BPA at ratios of 10, 30, and 50% of the total solvent volume to systematically investigate the effect of the BPA concentration. Following film formation, X-ray photoelectron spectroscopy (XPS) and Fourier transform infrared (FT-IR) analysis were performed and confirmed that BPA is removed after post processing (Figures S1 and S2).

**Figure 1:**
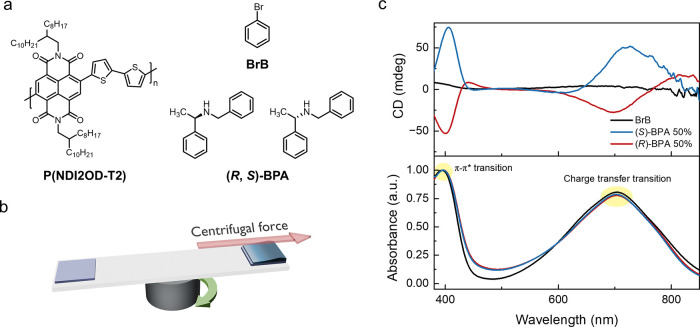
(a) Chemical structure of P­(NDI2OD-T2), (*R,S*)-(±)-*N*-benzyl-1-phenylethylamine (BPA), and bromobenzene (BrB). (b) Schematic of off-center spin coating. (c) Circular dichroism (CD) spectra and UV–vis spectra of P­(NDI2OD-T2) films of BrB (black), (*S*)-BPA 50% (blue), and (*R*)-BPA 50% (red).

UV–Visible absorption spectra for films with BrB (black), (*S*)-BPA 50% (blue), and (*R*)-BPA 50% (red) showed a π–π* transition peak^31^ at 395 nm for all three films and imply no significant changes to the dihedral angle between the NDI and T2 moieties during chiral induction (Figure 1c). In addition, the wavelength for peak maximum of the charge transfer transition is observed at 704 nm for BrB and at 703 nm for both (*S*)-BPA 50% and (*R*)-BPA 50% films, with a slight decrease in intensity. The change in intensity is attributed to disruption of the π–π stacking, which reduces intermolecular charge transfer. The degree of alignment was quantitatively evaluated using polarized UV–Vis absorption spectroscopy^32^ for films in (*S*)-BPA 10, 30, and 50%; the dichroism ratio (*A*_∥_/*A*_⊥_) increased from 1.04 to 1.14 and 1.35, respectively (Figure S3) and indicates that higher BPA concentrations improve molecular alignment. The dichroism ratios of BrB, (*S*)-BPA 50%, and (*R*)-BPA 50% films were 1.37, 1.35, and 1.27, respectively (Figure S4). These results imply that the chiral solvents provide a similar degree of alignment in films to that using conventional organic solvents that are capable of preaggregation.^33^ Figure 1c shows circular dichroism (CD) spectra of films prepared with (*S*)-BPA, 50% (blue); (*R*)-BPA, 50% (red); and without BPA (black). The mirror image response for films made with (*S*)- and (*R*)-BPA indicates the opposite chirality of the conjugated polymers, whereas films with just BrB display no chiroptical activity. Because the off-center spin coating method can result in anisotropic molecular alignment, potentially introducing linear dichroism and linear birefringence (LDLB) into the spectra, additional measurements were collected from both the front (β = 0°) and back (β = 180°) sides of the films and at two orthogonal orientations (θ = 0 and 90°) for the (*R*)-BPA 50% film (Figure S5). The CD spectra showed negligible differences when the films were flipped, whereas rotation resulted in observable changes in the CD intensity. These results suggest contributions from LDLB, as well as potentially imperfections with the photoelectric modulator.^34,35^ Henceforth, the front of two orthogonal film orientations were averaged to report the CD and g-factor. The CD values at 400 nm of the films increased with the enantiomeric excess (ee) determined by the (*S*)-BPA ratio (Table S1, Figure S6). The CD–ee plot exhibits a relatively rare anti-S-shaped, negative nonlinear effect, characterized by a steeper slope near −100% ee.^36,37^ Films prepared with 10, 30, and 50% (*S*)-BPA exhibit g-factors of 9.4 × 10^–5^, 8.6 × 10^–4^, and 2.0 × 10^–3^, respectively (Figure S7), indicating that the extent of chirality induction increases with chiral BPA concentration.

P­(NDI2OD-T2) possesses two chiral carbon atoms in its alkyl side chains (Figure S8a), and the dihedral angle between the NDI and T2 units has been reported to be approximately 47°.^38^ Our DFT calculations also revealed a comparable dihedral angle of 47° for the P­(NDI2OD-T2) monomer and a twisted molecular geometry in the side view of the trimer, indicating a pronounced backbone distortion (Figure S8b,c). Although the CD value of BrB is negligible compared to that of 50% BPA, chirality in P­(NDI2OD-T2) may still be induced through combined interactions between the backbone and side chains of the conjugated polymer mediated by the chiral solvent; however, the detailed mechanism of chiral induction and negative nonlinear effect remain unclear at present. Nevertheless, the observed dependences of the ee plot and the proportion of chiral solvent clearly demonstrate a BPA concentration-dependent enhancement of chirality induction in P­(NDI2OD-T2).

Atomic force microscopy (AFM) was used to analyze morphology, and the alignment was further evaluated (Figures S9 and S10). The films cast from BrB exhibited the typical P­(NDI2OD-T2) fibril morphology,^39^ whereas the films prepared with BPA showed larger fibril structures and higher root-mean-square (RMS) roughness. The orientational order parameter,^40^ calculated from AFM phase images, was 0.35 for BrB and 0.33 for both (*S*)-BPA 50% and (*R*)-BPA 50% films, further corroborating the polarized UV–vis results. In addition, polarized optical microscopy (POM) images show that fibrils in the BPA blended films were aligned along the direction of centrifugal force (Figure S11).

We performed scanning transmission electron microscopy (STEM) and scanning electron microscopy (SEM) to probe the alignment of the chiral conjugated polymer fibrils in detail. In films with (*S*)-BPA 50% and (*R*)-BPA 50%, the images reveal micrometer length scale fibrils with ∼100 nm width, possessing distinct twisted morphologies (Figures S12 and S13). For comparison, films prepared using only BrB as the solvent revealed the formation of small fibrils with thicknesses of a few nanometers and lengths of several tens of nanometers, consistent with previous reports on films cast from aromatic solvents.^41^ Collectively, these electron microscopy images show that chiral solvents lead to the formation of twisted fibrils in the P­(NDI2OD-T2) films.

### 2.2 Morphological Characteristics of Chiral Conjugated Polymers

To understand the effect of solvent on the morphological structure of P­(NDI2OD-T2) at the molecular level, we performed FT-IR spectroscopy on films made with BrB and (*S*)-BPA 10%, 30%, and 50%, respectively (Figure 2a). Note again that no signal associated with BPA, ca. 1100–1500 cm^–1^,^42,43^ is observed in the spectra, which implies complete removal of BPA. FT-IR analysis enables the investigation of backbone moiety orientation and conformational changes of the conjugated polymer.^44,45^ In the 1800–1600 cm^–1^ region, which primarily corresponds to the vibrational modes of the NDI2OD moiety, two characteristic bands (a and b) were analyzed (Figure 2b). Band a at 1705 cm^–1^, corresponds to the C = O symmetric stretching vibration and is mainly polarized perpendicular to the polymer backbone, whereas band b at 1666 cm^–1^, is attributed to the C = O asymmetric stretching vibration and is polarized along the polymer chain direction. Changes in the relative intensities of these two peaks reflect changes in the conformation and orientation of the polymer chain relative to the substrate (Figure 2d). The detailed calculation process of FT-IR spectra is provided in Section 4 of the Supporting Information. The average intensity ratio ⟨*R* ^b,a^⟩ serves as an indicator of the coplanarity of the NDI2OD unit. For BrB, ⟨*R* ^b,a^⟩ was approximately 1.16, but increased to 1.20, 1.20, and 1.23 for (*S*)-BPA 10, 30, and 50%, respectively (Figure 2e). The progressive increase in ⟨*R* ^b,a^⟩ with BPA concentration suggests that the NDI2OD moiety on the backbone becomes increasingly coplanar in conformation to the substrate. The 950–700 cm^–1^ region of the IR spectrum is mainly associated with the vibrational modes of the T2 unit, and again two main contributions are observed (c and d). The band *c* at 791 cm^–1^ corresponds to an out-of-plane C–H bond bending and the band *d* at 723.2 cm^–1^ corresponds to an in-plane C–S bond stretching (Figure 2c,f). The intensity ratio ⟨*R* ^d,c^⟩ reflects the planarization of the T2 unit (Figure 2g). In this case, ⟨*R* ^d,c^⟩ decreased from 0.93 for BrB to 0.91, 0.91, and 0.88 for (*S*)-BPA 10, 30, and 50%, respectively, and suggests that the T2 moiety on the backbone becomes less planar to the substrate. The ⟨*R* ^b,a^⟩ and ⟨*R* ^d,c^⟩ of (*R*)-BPA 50% are identical to (*S*)-BPA 50% (Tables S2 and S3). Notably, no significant peak shifts were observed for the C = O, C–H, and C–S vibrations as a function of the chiral solvent ratio, which indicates that the fundamental vibrational modes remain unchanged. The percentage change in ⟨*R* ^b,a^⟩ and ⟨*R* ^d,c^⟩ as a function of the chiral solvent concentration is approximately linear (Figure 2h) and indicates a progressive change in the planar conformational properties of the NDI2OD and T2 moieties upon chiral induction. The relative tilt of the backbone moiety with respect to the substrate can be estimated from the sum of ⟨*R* ^b,a^⟩ and ⟨*R* ^d,c^⟩. The higher sum indicates improved planarization, while a lower sum suggests an increased tilt. In our study, the sum of ⟨*R* ^b,a^⟩ and ⟨*R* ^d,c^⟩ is the same for all the films (ca. ∼2.1), i.e., independent of BPA concentration (Figure 2i). These results suggest that the fundamental structure and average dihedral angle between the NDI2OD and T2 units do not change and agree with no shift in the π–π* transition peak in the UV–vis spectra. Therefore, the changes in FT-IR intensity ratios are not attributed to changes in the planarization of individual moiety, but rather to the overall rotation of the conjugated polymer chains (Figure 2j). This collective polymer rotation induced by the chiral solvent is responsible for the formation of chiral fibrils in the P­(NDI2OD-T2) films.

**Figure 2:**
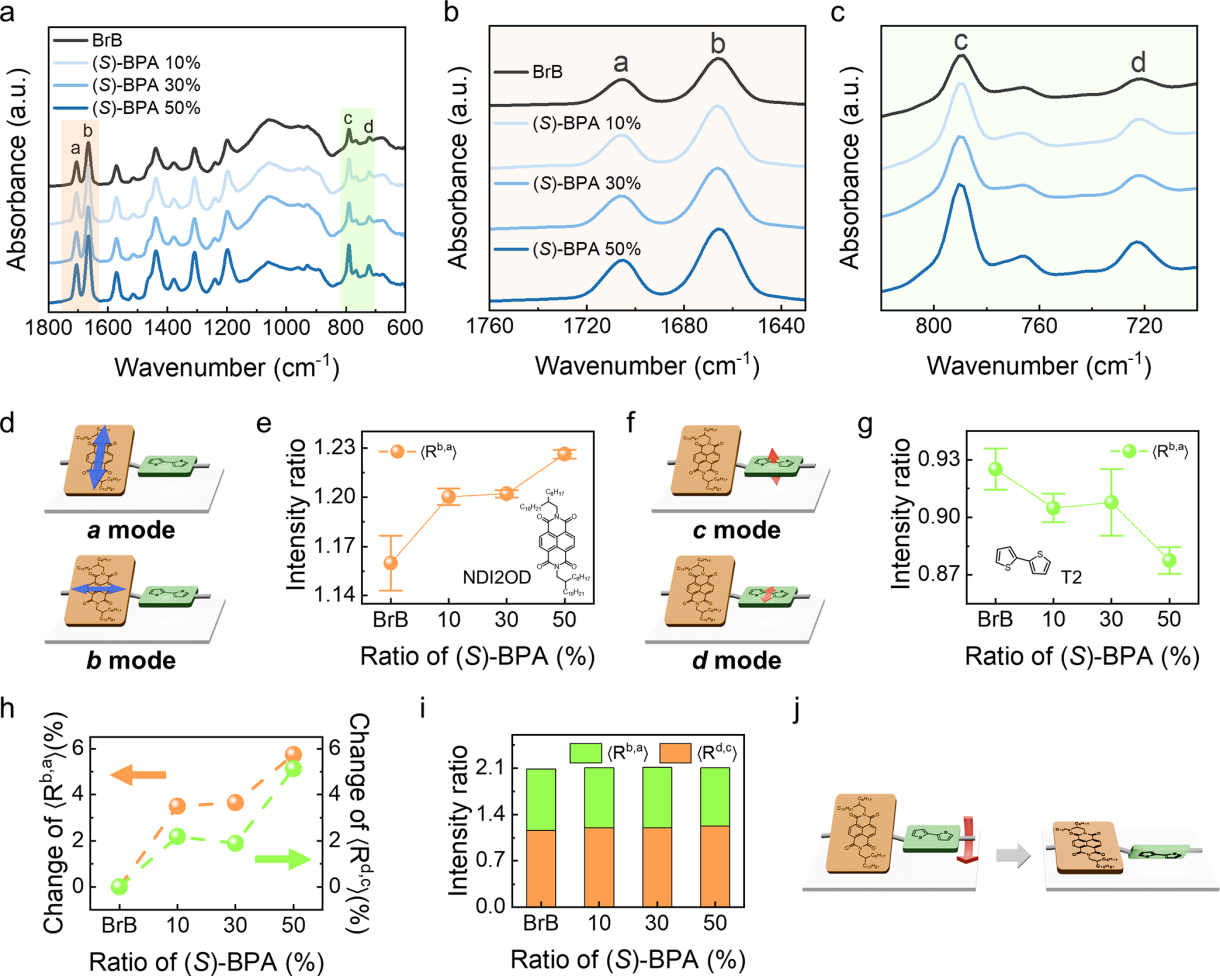
FT-IR spectra of P­(NDI2OD-T2) films corresponding to the ratio of (*S*)-BPA in (a) 1800–600 cm^–1^, (b) 1760–1630 cm^–1^, and (c) 820–700 cm^–1^ region, respectively. (d) Schematic of the a and b vibration modes of NDI2OD (arrows indicating the polarization directions of the backbone units). (e) Correlation between NDI2OD unit intensity ratio and <*R* ^b,a^> with standard deviation of (*S*)-BPA. (f) Schematic of the *c* and *d* vibration modes of T2. (g) Correlation between T2 unit intensity ratio and <*R* ^d,c^> with standard deviation of (*S*)-BPA. (h) Percentage of change of <*R* ^b,a^> and <*R* ^d,c^>, according to ratio of (*S*)-BPA. (i) Sum of the intensity ratio (<*R* ^b,a^> + <*R* ^d,c^>) according to the ratio of (*S*)-BPA. (j) Schematic of the overall rotation of P­(NDI2OD-T2) by BPA relative to the substrate.

To elucidate the crystallinity and fibril structure of P­(NDI2OD-T2) induced by the chiral solvent, we performed grazing incidence wide-angle X-ray scattering (GIWAXS) analysis on the films with the X-ray beam incident along the alignment direction (Figure 3a). For BrB films, the GIWAXS patterns exhibited (100), (200), and (300) peaks in the in-plane direction, which are indicative of lamellar stacking, as well as a pronounced (010) peak in the out-of-plane direction, corresponding to π–π stacking (Figure 3b). Additional chain backbone repeating peaks (001), (002), and (004) were also observed. These peaks confirm the face-on orientation of P­(NDI2OD-T2).^46^

**Figure 3:**
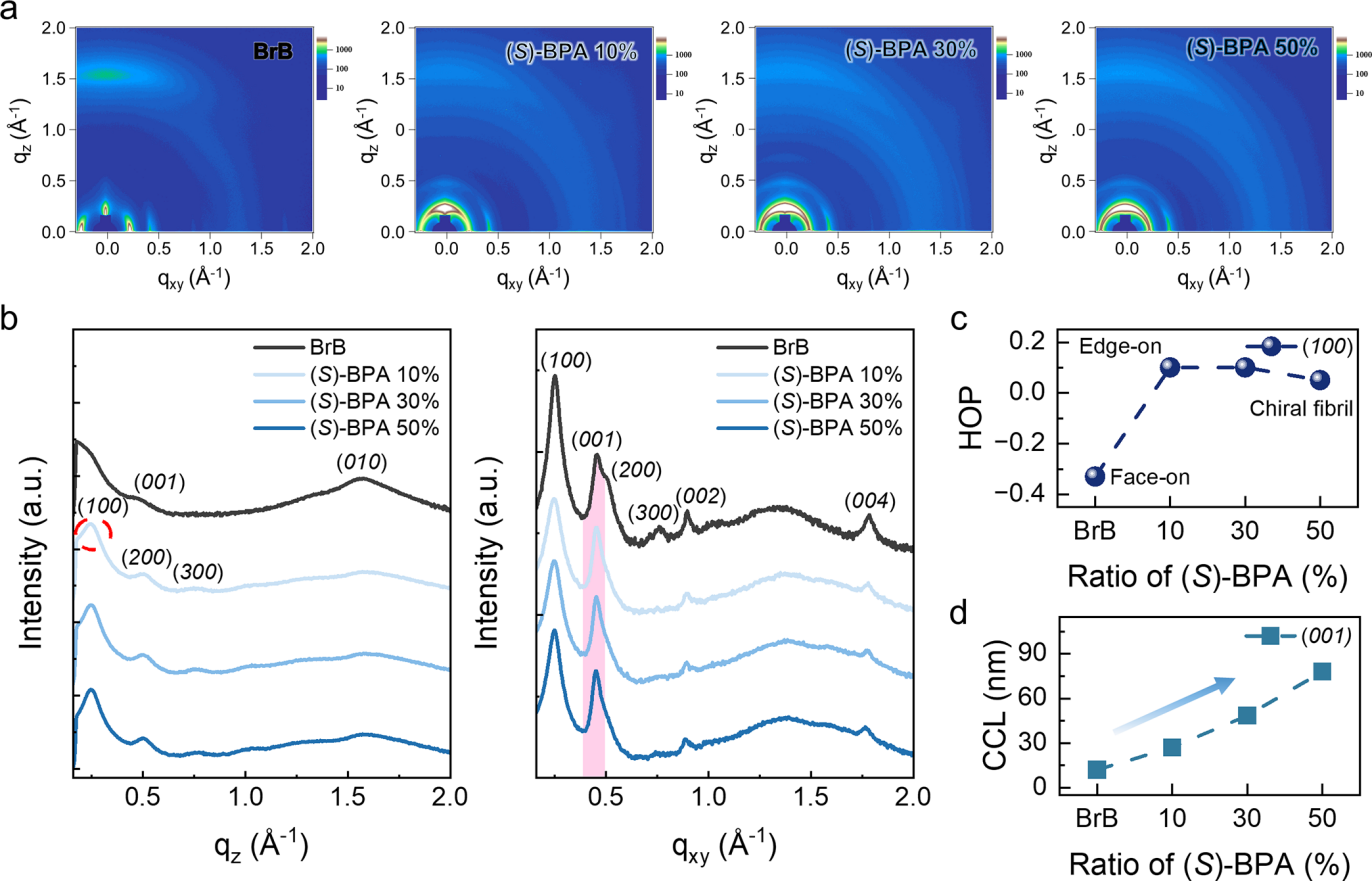
(a) 2D-GIWAXS profile images depending on (*S*)-BPA ratio. (b) Linecut graph with P­(NDI2OD-T2) peaks assigned. (c) Herman’s orientation parameters of (100) (d) CCL in in-plane at the (001) crystal peaks (pink area on in-plane linecut graph).

The introduction of the chiral solvent resulted in significant changes in the lamellar stacking. The (*S*)-BPA 10% showed new (100), (200), and (300) peaks in the out-of-plane direction, while the (200) and (300) peaks disappeared in the in-plane direction. These results show that the chiral solvent changes the lamellar stacking of the conjugated polymers and is responsible for imparting chirality into the films. To quantify changes in lamellar stacking orientation, we calculated Herman’s orientation parameter (HOP), which is based on the azimuthal dependence of the (100) scattering intensity.^47,48^ The HOP ranges from 1.0 (edge-on) to −0.5 (face-on), with 0.0 indicating no preferential orientation. For the BrB film, the HOP was −0.33, displaying face-on orientation (Figure 3c and Table S6). Conversely, the HOP increased to 0.10, indicating a shift toward edge-on lamellar stacking in (*S*)-BPA 10 and 30%. At (*S*)-BPA 50%, the HOP decreased to 0.05, i.e., became more randomly oriented. Additionally, we compared the intensity ratio of the (100) peaks in the in-plane to the out-of-plane directions (Figure S14). This ratio increased from 0.86 to 0.88 and 0.92 for (*S*)-BPA 10, 30, and 50%, respectively, with no significant change in lamellar stacking distance (Table S4a,b). Analysis of the (100) peak crystalline coherence length (CCL), calculated from the full width at half-maximum (fwhm), showed that the in-plane to out-of-plane CCL ratio decreased from 2.14 at (*S*)-BPA 10% to 1.04 at (*S*)-BPA 50% (Table S5). These findings support isotropic lamellar stacking and crystalline development, which ultimately result in the formation of chiral conjugated polymers. The π–π stacking distance from the in-plane (010) peak decreased slightly from 4.00 Å to 3.94 Å, 3.93 Å, and 3.94 Å for BrB, (*S*)-BPA 10, 30, and 50%, respectively, which we attribute to nanofibril formation, consistent with other works.^49^ However, the corresponding CCL decreased significantly from 7.05 nm on BrB to 2.92, 2.73, and 2.99 nm with increasing (*S*)-BPA (Table S4c), indicating that nanofibrils do not extend along the π–π stacking direction. The reduction in the CCL of π–π stacking is consistent with the UV–Vis absorption results, specifically the diminished intensity of the charge transfer transition in the (*S*)-BPA 50%. For the HOP of (010), the values decrease from 0.26 in BrB to 0.09 in (*S*)-BPA 50% (Table S6), respectively, and confirm an orientational transition from face-on to isotropic π–π stacking. The HOP of (010) parallels the (100) HOP behavior, demonstrating that the transition to isotropic lamellar stacking simultaneously induces isotropic π–π stacking. The CCL along the (001) direction increased markedly and linearly, from 11.92 to 26.98, 48.43, and 78.06 nm for BrB, (*S*)-BPA 10, 30, and 50%, respectively (Figure 3d). The substantial increase in CCL of the (001) peaks suggests the formation of a chiral fibril structure along the (001) direction. The GIWAXS analysis of films blending with (*R*)-BPA exhibited the same trends as those observed with (*S*)-BPA in Section 5 of the Supporting Information. In conclusion, through GIWAXS analysis, the chiral solvent induces isotropic lamellar stacking of the conjugated polymer and crystallization along the (001) direction, leading to the formation of chiral fibrils.

In summary, chirality was successfully induced in the n-type conjugated polymer P­(NDI2OD-T2) using chiral solvents. At the molecular level, the polymer backbone undergoes an overall rotation, while at the crystalline level, the lamellar stacking becomes isotropic with enhanced crystallinity along the (001) backbone direction (Scheme 1). These structural changes promote the formation of chiral fibers, and we suggest a chiral fibril structure.

**Scheme 1:**
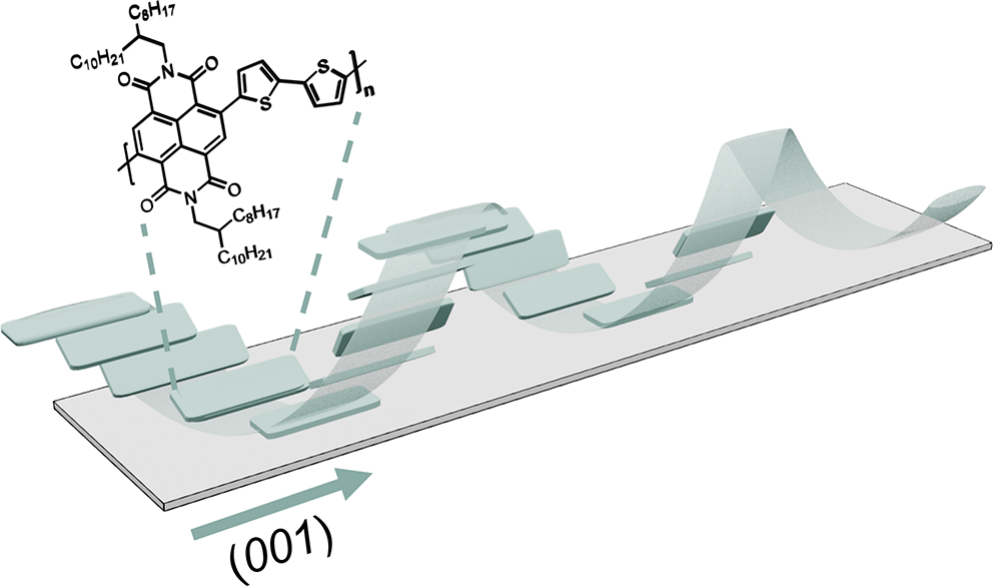
Schematic Illustration of Chiral-Induced P­(NDI2OD-T2) by BPA Through Reorganization of Crystalline (Green Domain) in Films

### 2.3 Magneto Field-Effect Transistors

To investigate the CISS properties of the chiral conjugated polymer films, bottom-gate bottom-contact magneto field-effect transistor (mFET) devices were fabricated (Figures 4a and S16). The conjugated polymer fibrils were aligned parallel to the current flow direction through an off-center spin coating method. The n-type conjugated polymers are typically degraded under continuous measurements in air due to oxidative processes (Figure S17).^50^ Consequently, the achiral BrB films exhibit instability during continuous measurements and show an electron mobility of 6.8 × 10^–7^ cm^2^V^–1^s^–1^ and a threshold voltage of 30.4 V in the first measurement without an external magnetic field (Table S8). In addition, the electrical characteristics of BrB films are not improved by an external magnetic field.

**Figure 4:**
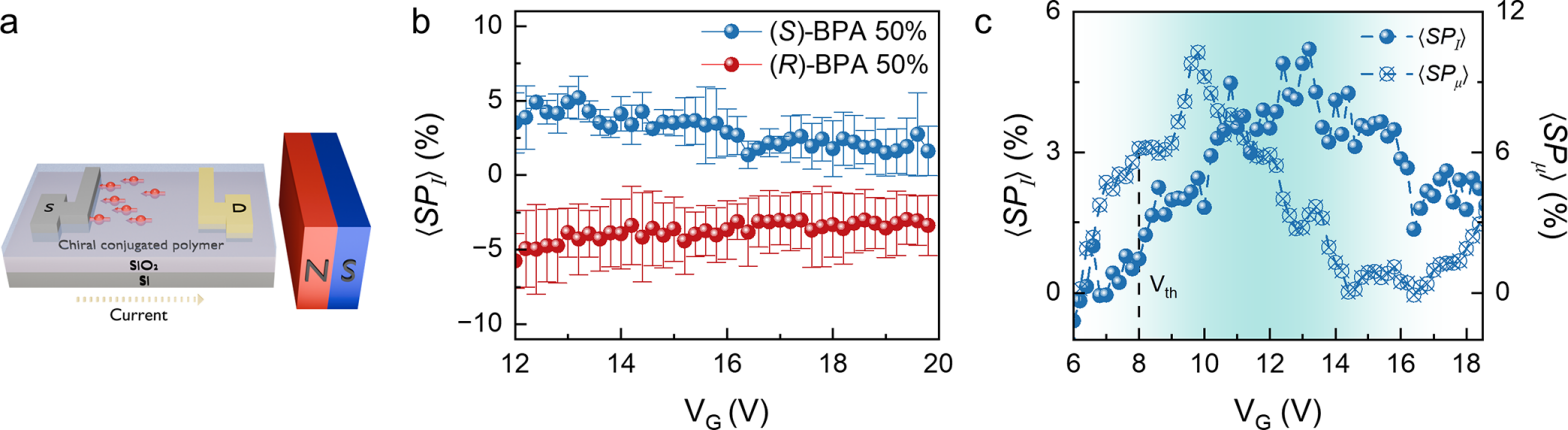
(a) Schematic of the magneto field-effect transistor. (b) Average of spin polarization current with standard deviation of (*S*)-BPA 50%, and (*R*)-BPA 50%. (c) Average of spin polarization current (⟨*SP*_*I*_⟩ left axis) and mobility (⟨*SP*_μ_⟩ right axis) of (*S*)-BPA 50%.

Notably, the chiral conjugated polymer films of (*S*)-BPA 50% and (*R*)-BPA 50% exhibit higher transistor performance in the absence of a magnetic field than the BrB films, with electron mobilities of 1.7 × 10^–4^ and 4.8 × 10^–5^ cm^2^V^–1^s^–1^ and threshold voltages of 10 and 18.5 V, respectively. These enhancements are attributed to nanofiber structures induced by the chiral solvent, which facilitate long-range charge transport.^51^ In contrast to the continuous measurements without a magnetic field, the application of an external magnetic field consistently enhances the current in the chiral conjugated films, regardless of whether the north (*N*) or south (*S*) pole is applied (Figure S19). The average mobility measured under the *N* pole for the (*S*)-BPA 50% was 2.1 × 10^–4^ cm^2^V^–1^s^–1^ (Table S8), representing a 23% increase compared to that without a magnetic field. For the (*R*)-BPA 50%, the mobility increased by 69%, reaching 8.1 × 10^–5^ cm^2^V^–1^s^–1^.

Furthermore, the asymmetry in current with an *N* and *S* applied field was quantified as an average spin polarization current (⟨*SP*_*I*_⟩) as described in the Experimental section. Note that the average spin polarization was obtained by averaging current measurements from multiple independently fabricated devices to account for the potential measurement variability. Above the threshold voltage, the ⟨*SP*_*I*_⟩ reached a maximum of 7.5% for the (*S*)-BPA 50 and −11.6% for the (*R*)-BPA 50% (Figure 4a), with an average of 5.2% for the (*S*)-BPA 50 and −5.0% for the (*R*)-BPA 50%. The average spin polarization mobility (⟨*SP*_μ_⟩) was defined analogously to spin polarization current by substituting the gate-dependent mobility for current in the corresponding equation. At the gate voltage of 10 V, the ⟨*SP*_μ_⟩ of (*S*)-BPA 50% and (*R*)-BPA 50% reached 11.7 and −8.6%, respectively (Figure S20a). The opposite ⟨*SP*_*I*_⟩ and ⟨*SP*_μ_⟩ for the two enantiomorphs are consistent with a CISS phenomenon. For both films, the CISS response increases near the threshold voltage but decreases at higher gate voltages (Figures 4c and S20b), suggesting that CISS is most pronounced near the onset of conduction. At higher gate voltages, it is possible that additional achiral transport channels and/or scattering of polarized spins occur that diminish the observed polarizations.

## 3 Conclusion

In this study, we successfully induced chirality in n-type conjugated polymer P­(NDI2OD-T2) using chiral solvents derived from phenylethylamine. The polymers form aligned chiral fibrils without a residual chiral solvent. The resulting chiral conjugated polymers were incorporated into magneto field-effect transistor devices, which showed improved transistor performance compared to their achiral counterpart. In addition, a robust asymmetry in both current and mobility with orientation of an external magnetic field was observed, and it displayed a mirror image relationship for the polymer enantiomorphs. Collectively, these findings deepen the understanding of chiral induction through morphology in achiral conjugated polymers and demonstrate spin-selective transport in organic semiconductors, paving the way toward rational design of high-performance spin transistors for next-generation spintronic applications.

## 4 Experimental Section

### 4.1 Materials

The P­(NDI2OD-T2) (MW = 130 kDa, PDI ∼ 2.5) was purchased from 1-Material, bromobenzene was purchased from Sigma-Aldrich, and (*R*,*S*)-(±)-*N*-benzyl-1-phenylethylamine was purchased from TCI.

### 4.2 Film Characterization

Before the fabrication of films on glass and silicon substrates, the substrates were cleaned in an ultrasonic bath containing acetone and isopropanol, then exposed to UV ozone for 5 min. Deposition solutions were prepared by dissolving P­(NDI2OD-T2) (10 mg/mL) in a blend of bromobenzene and (*R*,*S*)-(±)-*N*-benzyl-1-phenylethylamine at the specified volume ratios. The blended solution was stirred overnight at 65 °C on a hot plate. The solutions were then off-center spin-coated onto the substrates at 300 rpm for 60 s and accelerated for 10 s under a N_2_ atmosphere. The films are dried in a vacuum chamber for 2 days following spin coating.

CD spectra were measured in the film state by using a JASCO J-810 spectrophotometer at room temperature. UV–vis absorption spectra were collected using a Cary 5000 (PerkinElmer). AFM analysis was performed in noncontact mode using XE-100 (Park Systems). POM images were captured using a Carl Zeiss Axio A1 microscope in the polarized light mode. XPS data were collected on silicon substrates using a NEXSA instrument (Thermo Fisher Scientific). TEM and STEM analyses were performed using a Tecnai G2 F30 S-Twin 300 kV instrument (FEI). Cross-sectional SEM images on silicon substrates were obtained using a Verios 5 UC. (Thermo Fisher Scientific). FT-IR spectra were obtained using a Vertex 70v instrument (Bruker). The optimized conformation of P­(NDI2OD-T2) was calculated via density functional theory (DFT) with B3LYP/6–31G­(d,p) in Gaussian 16. GIWAXS of films coated on silicon substrates was performed under vacuum at room temperature using an 11.06 keV X-ray from a 9A beamline at the Pohang Accelerator Laboratory (PAL).

### 4.3 Field-Effect Transistor Fabrication

A 285 nm thick SiO_2_ (*C_i_* = 12.1 nF cm^–2^) silicon wafer substrate was used to fabricate the FET devices in a coplanar (bottom gate/bottom contact) structure as follows: Ni/Au (50/10 nm) for source and Ti/Au (10/50 nm) for drain electrodes were fabricated using a conventional lift-off photolithography process. The substrates were cleaned sequentially in acetone and isopropanol baths, followed by UV-ozone treatment for 5 min. The channel width/length was 3.0 mm/10 μm.

### 4.4 Magneto Field-Effect Transistor Characterization

The transistor performances were carried out using a Keithley 2636B instrument in the air. The μ and *V*_Th_ values were calculated in the saturation regime (*V*_DS_ = 20 V) of transfer curves according to the following equation

1 $$I_{D}=\frac{W}{L}\mu_{\mathrm{FET}}C_{i}{\left(V_{G}-V_{\mathrm{Th}}\right)}^{2}$$

2 $$\mu =\frac{2L}{WC_{i}}{\left(\frac{d\sqrt{I_{D}}}{dV_{G}}\right)}^{2}$$

where *W* and *L* are the channel width and length, respectively; *V*_G_ and *V*_Th_ are the gate and threshold voltages, respectively; and *C_i_* is the capacitance per unit area of the dielectric layer.

The spin polarization current (*SP*_*I*_), mobility (*SP*_μ_), and their respective averages are as follows

3 $${\mathrm{SP}}_{I}=\frac{\sum_{i=2}^{M}I_{N,i}-\sum_{i=2}^{M}I_{S,i}}{\sum_{i=2}^{M}I_{N,i}+\sum_{i=2}^{M}I_{S,i}}\times 100\%$$

4 $$\left\langle {\mathrm{SP}}_{I}\right\rangle =\frac{1}{N}\sum_{j=1}^{N}{\mathrm{SP}}_{I}^{\left(j\right)}$$

5 $${\mathrm{SP}}_{\mu }=\frac{\sum_{i=2}^{M}\mu_{N,i}-\sum_{i=2}^{M}\mu_{S,i}}{\sum_{i=2}^{M}\mu_{N,i}+\sum_{i=2}^{M}\mu_{S,i}}\times 100\%$$

6 $$\left\langle {\mathrm{SP}}_{\mu }\right\rangle =\frac{1}{N}\sum_{j=1}^{N}{\mathrm{SP}}_{\mu }^{\left(j\right)}$$

In the equation (eq 3), *I*_*N*,*i*_ and *I*_*S*,*i*_ represent the currents measured during the *i*th measurement with the *N* pole and *S* pole applied, respectively, while μ_*N*,*i*_ and μ_*S*,*i*_ (eq 5) denote the gate-dependence mobility measured under the same conditions. For each device, *M* represents the total number of measurements taken for each pole, and the calculation begins from *i* = 2 to exclude the artificial effect caused by the measurement without a magnetic field.

The total number of devices is denoted by *N* (eqs 4 and 6), and *SP*_*I*_^(*j*)^ and *SP*_μ_^(*j*)^ indicate the spin polarization current and spin polarization mobility measured from the *j*th device, respectively. The current was measured alternately at the *N* and *S* poles. The magnetic field was approximately 200 mT.

## Supplementary Material



## Notes

Discussion of residual chiral solvent by XPS and FT-IR; alignment and chirality analysis by UV–vis and CD; DFT calculations; chiral fibril images by AFM, SEM, TEM, and STEM; equation for FT-IR analysis; d-spacing peaks and crystal coherence length values from GIWAXS; schematic of magneto field-effect transistors under varying magnetic field conditions; transfer and output curves with and without magnetic field; and spin polarization current and mobility of several devices (PDF)
