# Supplementary material for: Inducing Chirality in n‑Type Conjugated Polymers by Chiral Solvents for the Development of Spin Transistors
Source: ACS Appl Mater Interfaces. 2026 Apr 9;18(15):22225–33. doi: 10.1021/acsami.6c02690 (PMC13107376; doi:10.1021/acsami.6c02690)
Supplement: Supplementary file 1 [file am6c02690_si_001.pdf]

Supporting Information

Inducing Chirality in n-Type Conjugated Polymers by Chiral Solvents for the Development of Spin Transistors

Jeongwoo Beak [a], Yina Moon [a,b], Justin J. O'Neil [c], Dongseong Yang [d], Brian P. Bloom [c], Minwoo Lee [a], Geon Chang Song [e], Yunseul Kim [a,f], David H. Waldeck [c], Dong-Yu Kim [a*]*

[a] School of Materials Science and Engineering (SMSE), Research Institute for Solar and Sustainable Energies (RISE), Gwangju Institute of Science and Technology (GIST), Gwangju 61005, Republic of Korea

[b] Department of Chemistry, Purdue University, 560 Oval Drive, West Lafayette, IN, USA

[c] Department of Chemistry, University of Pittsburgh, 219 Parkman Avenue, Pittsburgh, Pennsylvania, 15260, United States

[d] User Convenience Technology R&D Department, Korea Institute of Industrial Technology (KITECH), Ansan-si, 15588, Republic of Korea

[e] Division of Advanced Materials, Korea Research Institute of Chemical Technology (KRICT), 141 Gajeong-ro, Yuseong-gu, Daejeon 34114, Republic of Korea

[f] Andlinger Center for Energy and the Environment, Princeton university, Princeton, NJ, 08540, United States

* E-mail: kimdy@gist.ac.kr

* E-mail: dave@pitt.edu

Table of Contents

Section 1. Analysis of residual chiral solvent

Section 2. Optical properties of chiral conjugated polymer films

Section 3. Chiral induction sites on P(NDI2OD-T2)

Section 4. Microscopy image analysis

Section 5. FT-IR analysis of NDI and T2 moiety in P(NDI2OD-T2)

Section 6. Crystallinity of chiral conjugated polymer

Section 7. Magneto field-effect transistor

Section 1. Residual chiral solvents

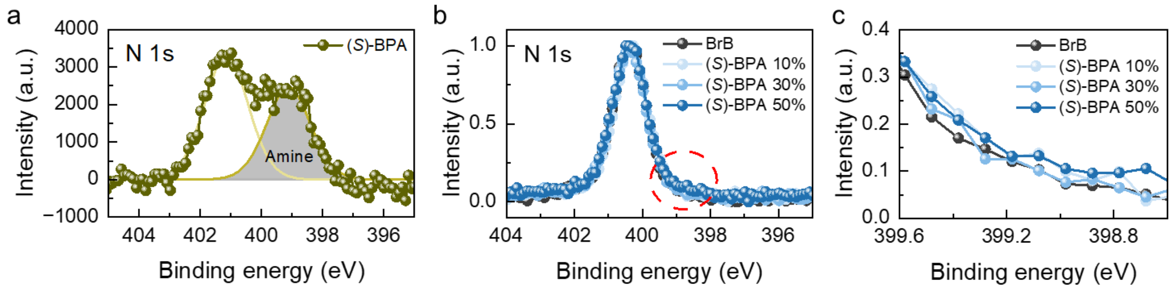

Figure S1. High resolution N1s X-ray Photoelectron spectra for a) pure (*S*)-BPA, and b) films of P(NDI2OD-T2) dissolved in BrB with (*S*)-BPA at various ratios following vacuum drying. Panel c) shows the tail of the spectra corresponding to the red circled region for the data depicted in panel b.

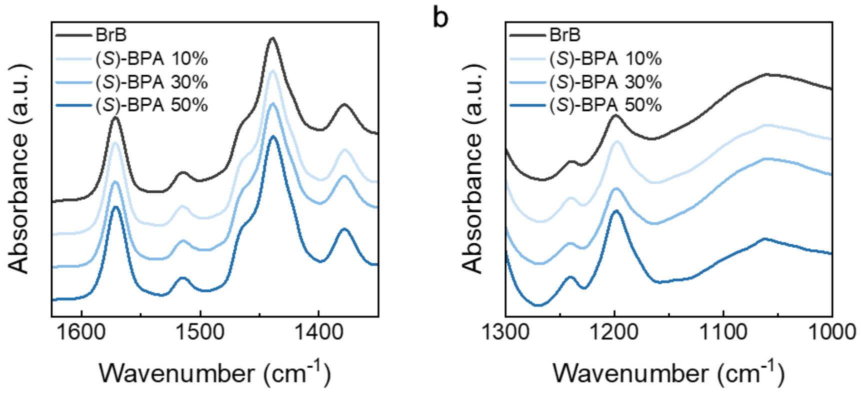

Figure S2. FT-IR spectra of P(NDI2OD-T2) films corresponding to the ratio of (*S*)-BPA in a)1650-1300 cm^{-1}, b) 1300-1000 cm^{-1}.

XPS analysis demonstrates that pristine BPA exhibits a characteristic amine N 1s peak at 399 eV (Figure S1a). However, this peak is not detected in the dried films at any (*S*)-BPA condition (Figure S1b). The magnified spectral region corresponding to the amine group (Figure S1c) confirms the absence of any detectable N 1s signal at 399 eV. FT-IR spectroscopy provides additional evidence for BPA removal. The characteristic N–H bending vibrations (1700–1400 cm^{-1}) and C–N stretching vibrations (1300–1000 cm^{-1}) of BPA are expected if residual amine molecules remain in the films. However, when comparing pristine P(NDI2OD-T2) films with BPA-treated films following drying, no peak shifts or additional peaks associated with BPA are observed in either spectral region (Figure S2a, b).

Section 2. Optical properties of chiral conjugated polymer films

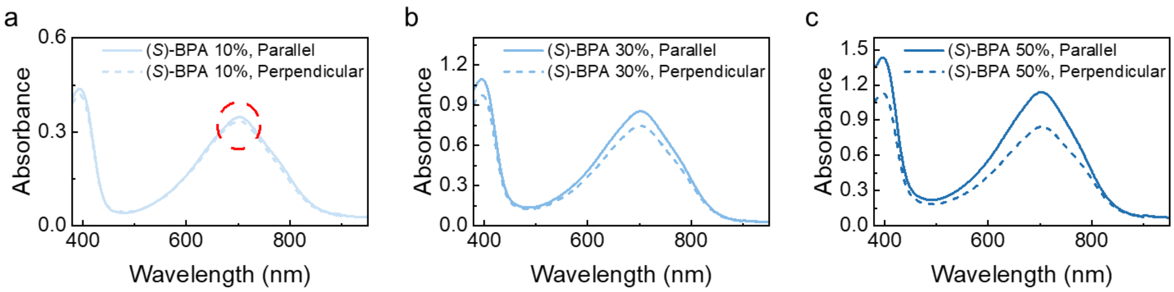

Figure S3. Polarized UV-Vis spectra measured in parallel and perpendicular directions of a) (*S*)-BPA 10%, b) 30%, and c) 50% (Calculated on dichroism ratio ($A_{\parallel}/A_{\perp}$) by max peak of red circle).

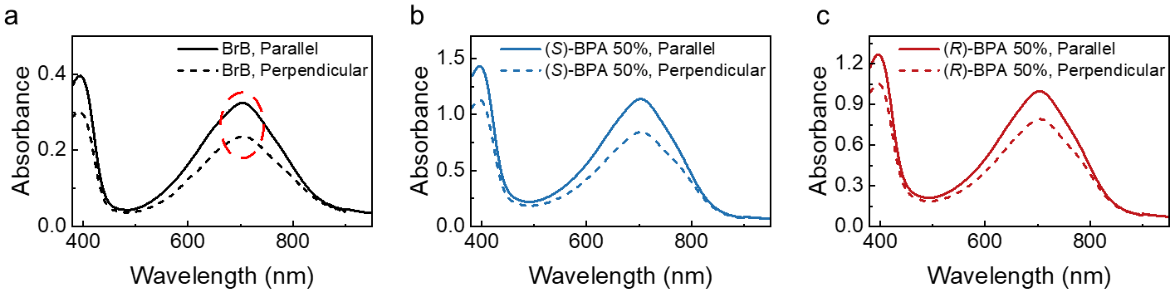

Figure S4. Polarized UV-Vis spectra measured in parallel and perpendicular directions of a) BrB, b) (*S*)-BPA 50%, and c) (*R*)-BPA 50% (Calculated on dichroism ratio ($A_{\parallel}/A_{\perp}$) by max peak of red circle).

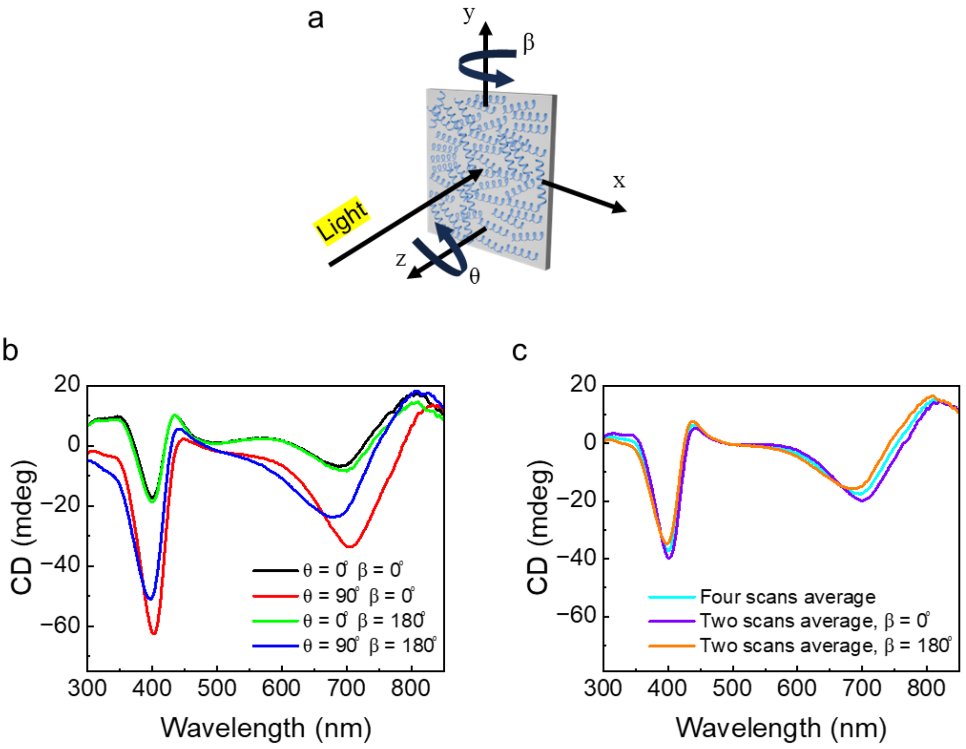

Figure S5. a) Schematic of the CD four-scan method, including in-plane rotation angle (θ) and out-of-plane rotation angle (β). b) CD spectra of four scans at different angles of (*R*)-BPA 50%. c) CD spectra of four scans average and the two scan averages when β is 0° and 180° of (*R*)-BPA 50%.

Equation of enantiomeric excess

$$ee = \frac{R - S}{R + S} \times 100\% \qquad (S1)$$

$$R, S = \text{Solvent volume ratio of } (R)\text{-}, (S)\text{-BPA, respectively.}$$

Table S1. Volume ratio of solution for enantiomeric excess of films.

ee (%)	Volume ratio in solution (%) BrB	(*S*)-BPA	(*R*)-BPA
0	50	25	25
-50	50	37.5	12.5
-100	50	50	0

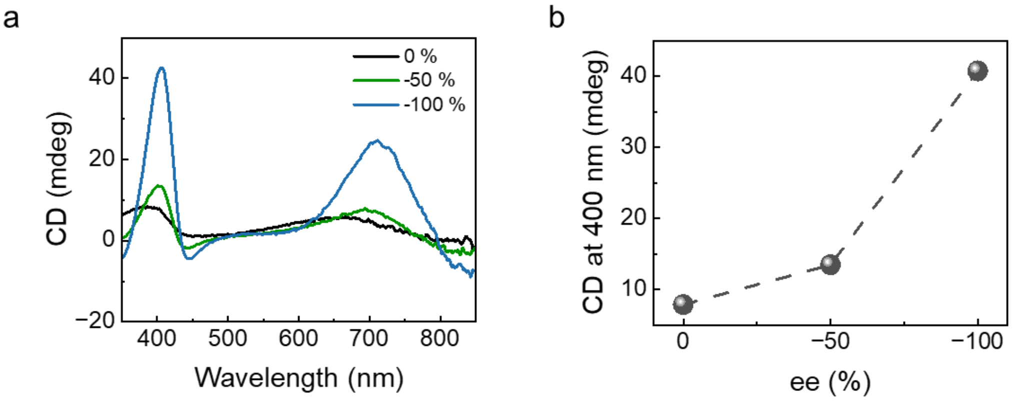

Figure S6. a) CD spectra of 0, -50, -100% ee, respectively. b) enantiomeric excess (ee) curve.

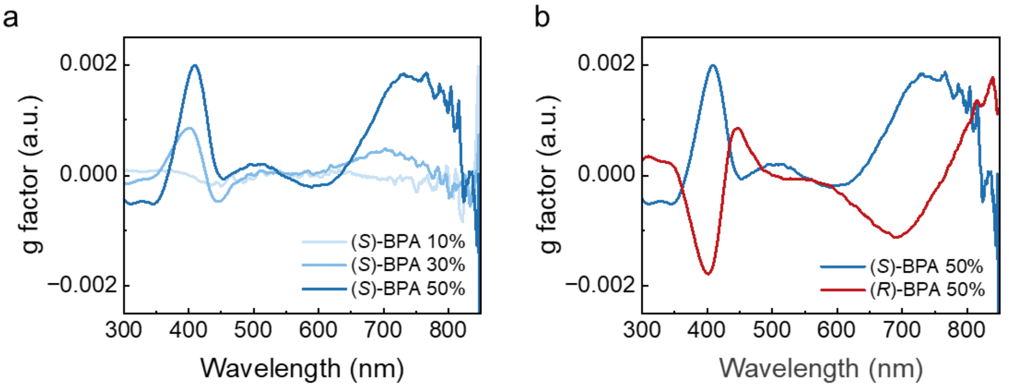

Figure S7. a) g factor based on the ratio of (*S*)-BPA. b) g factor of (*S*)-BPA 50% and (*R*)-BPA 50% on films.

Section 3. Chiral induction sites on P(NDI2OD-T2)

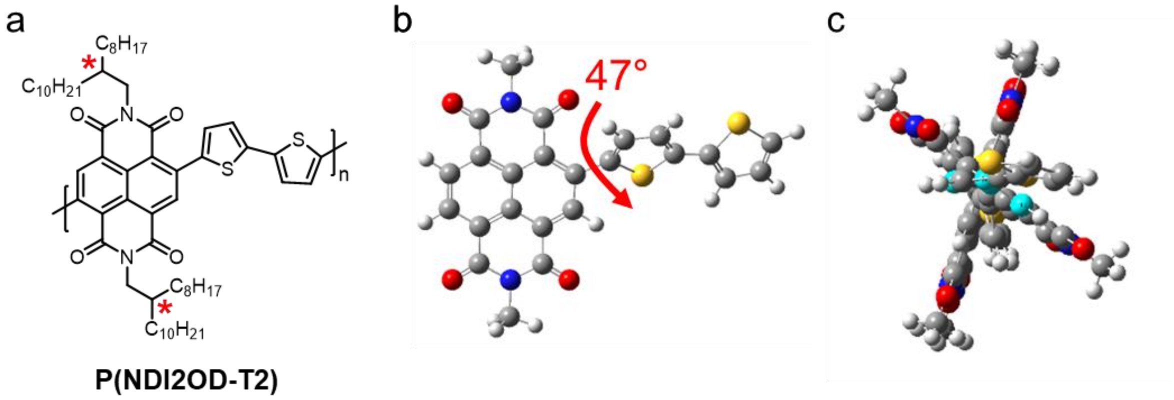

Figure S8. a) Chemical structure of P(NDI2OD-T2) with two chiral carbon atoms lab eled with red asterisks. b) DFT-optimized geometry of the P(NDI2OD-T2) monomer. c) Viewed from the side of a trimer P(NDI2OD-T2) model.

Section 4. Microscopy image analysis

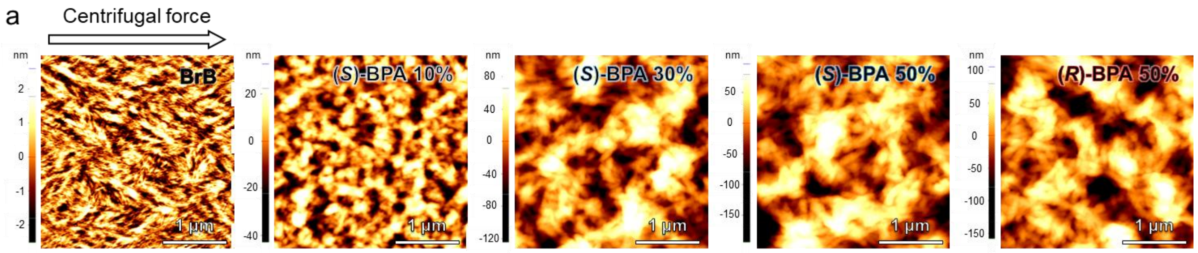

b

	(*S*)-BPA				(*R*)-BPA
BPA ratio (%)	0	10	30	50	50
RMS (nm)	0.91	11.4	33.4	40.5	40.4

Figure S9. (a) AFM topology images via chiral solvent ratio. b) Root mean square (RMS) of AFM topology by BPA ratios.

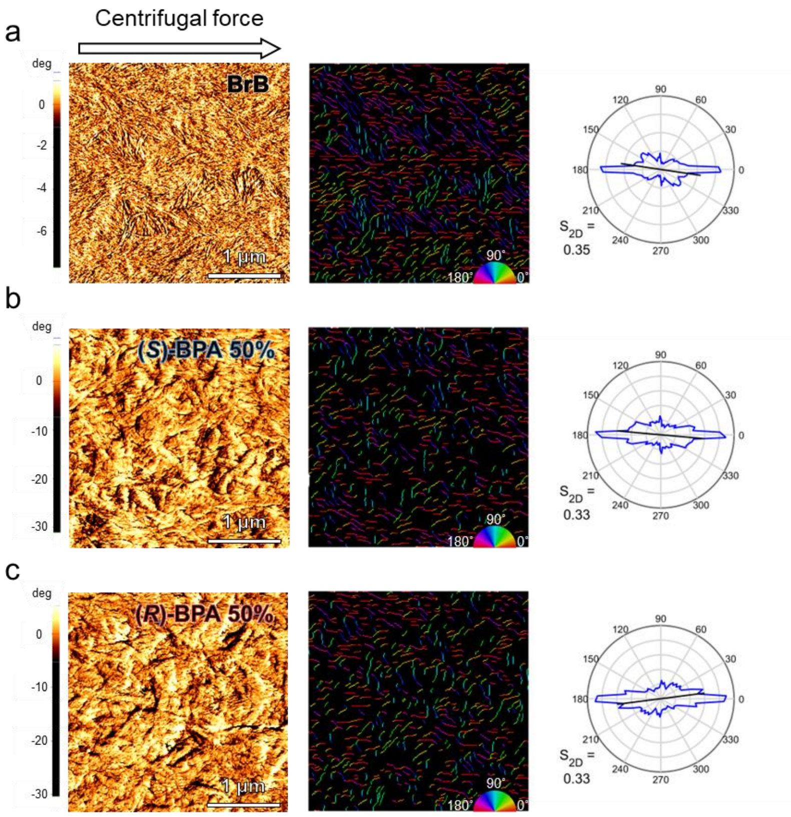

Figure S10. AFM NCM-phase images with orientation maps and orientation parameter (S_{2D}) of a) BrB, b) (*S*)-BPA 50%, and c) (*R*)-BPA 50%.

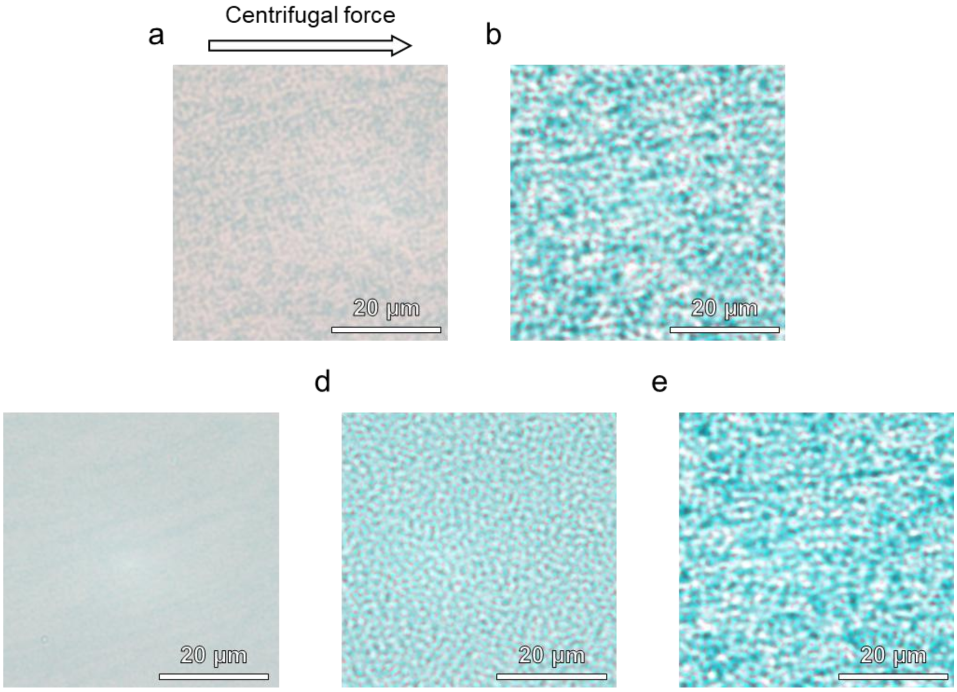

Figure S11. Polarized optical microscopy (POM) film images of a) BrB, b) (*R*)-BPA 50%, c) (*S*)-BPA 10%, d) (*S*)-BPA 30%, and e) (*S*)-BPA 50%.

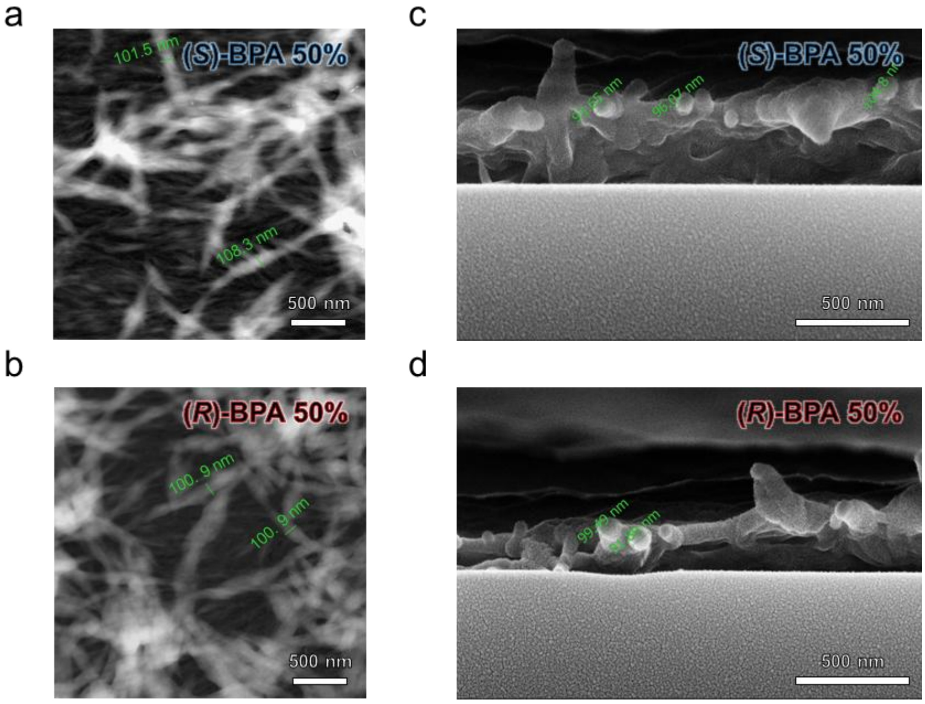

Figure S12. STEM images of a) (*S*)-BPA 50%, and b) (*R*)-BPA 50%, and cross-section of SEM images of c) (*S*)-BPA 50%, and d) (*R*)-BPA 50%.

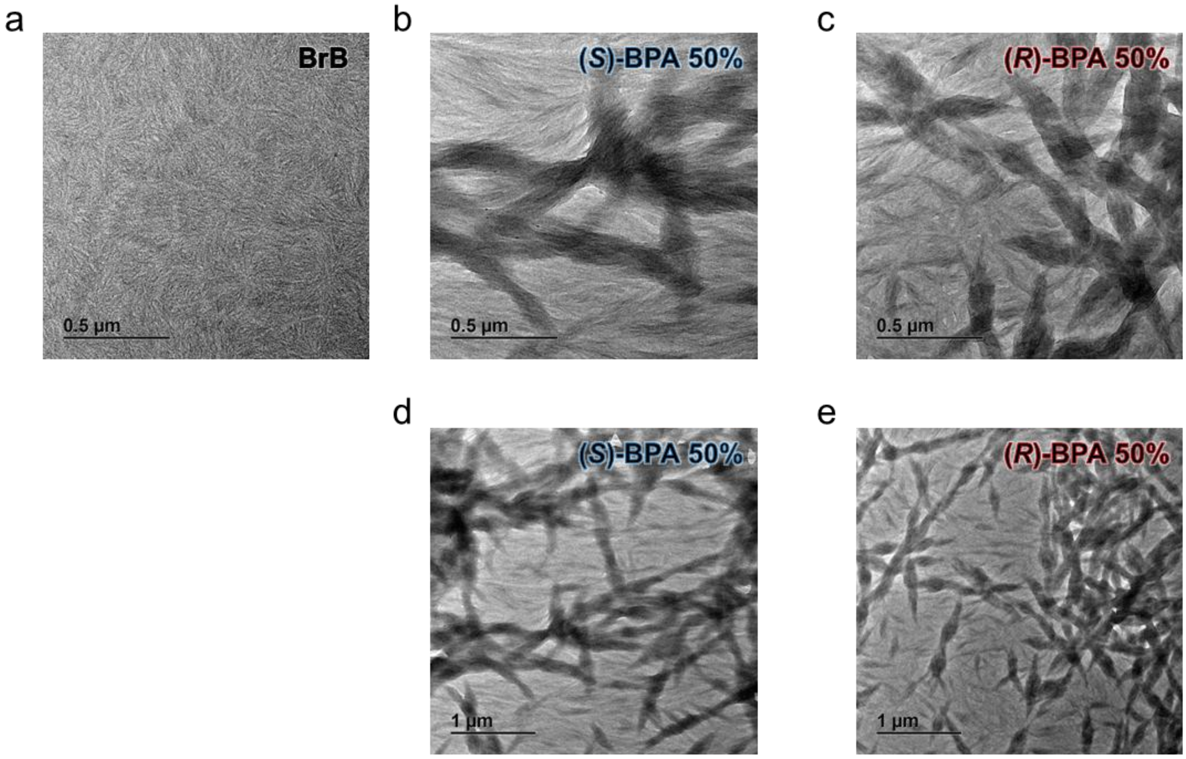

Figure S13. Relatively high magnification TEM images of a) BrB, b) (*S*)-BPA 50%, and c) (*R*)-BPA 50%. Lower magnification TEM images of d) (*S*)-BPA 50%, and e) (*R*)-BPA 50%.

Section 5. FT-IR analysis of NDI and T2 moiety in P(NDI2OD-T2)

The intensity ratio $R^{b,a}$ and $R^{d,c}$ are calculated by using the intensity of the NDI and T2 peaks in the FT-IR spectra[1,2] according to the flowing equations:

$$R^{b,a} = \frac{I^b}{I^a} \ or \ R^{d,c} = \frac{I^d}{I^c} \quad (S2)$$

$$R = R^{b,a} \ or \ R^{d,c}$$

$$N = number\ of\ samples$$

$$Average\ of\ R = \langle R \rangle = \frac{1}{N}\sum_{j=1}^{N} R \quad (S3)$$

$$Change\ of\ \langle R \rangle = \frac{\langle R^{BPA} \rangle - \langle R^{BrB} \rangle}{\langle R^{BrB} \rangle} \times 100\% \quad (S4)$$

Figure 2e and 2g show the average of $\langle R^{b,a} \rangle$, and $\langle R^{d,c} \rangle$ (eq S3), while Figure 2h shows change of $\langle R \rangle$ (eq S4).

Table S2. The $R^{b,a}$ values by samples according to the ratio of BPA.

$R^{b,a}$	BrB	(*S*)-BPA 10%	(*S*)-BPA 30%	(*S*)-BPA 50%	(*R*)-BPA 50%
Sample 1	1.17	1.19	1.20	1.23	1.23
Sample 2	1.17	1.20	1.20	1.22	1.22
Sample 3	1.14	1.20	1.20	1.23	1.24
Average	1.16	1.20	1.20	1.23	1.23

Table S3. The $R^{d,c}$ values by samples according to the ratio of BPA.

$R^{d,c}$	BrB	(*S*)-BPA 10%	(*S*)-BPA 30%	(*S*)-BPA 50%	(*R*)-BPA 50%
Sample 1	0.91	0.91	0.93	0.88	0.87
Sample 2	0.93	0.90	0.90	0.87	0.87
Sample 3	0.94	0.90	0.90	0.88	0.89
Average	0.93	0.91	0.91	0.88	0.88

Section 6. Crystallinity of chiral conjugated polymer

The CCL values were extracted using the standard Scherrer equation.[3,4]

$$CCL = \frac{K\lambda}{\beta \cos\theta} \qquad (S5)$$

where K = 0.9, λ is the X-ray wavelength (11.06 keV), β is the full width at half maximum (FWHM) in radians, and θ is the Bragg angle derived from the peak position.

Table S4. d-spacing peaks for a) out-of-plane, b) in-plane, and c) crystalline coherence length (CCL) as a function of (*S*)-BPA ratio.

a

(Å)	Out-of-plane (OOP)				
	(100)	*(001)*	*(200)*	*(300)*	*(010)*
BrB	-	13.93	-	-	4.00
(*S*)-BPA 10%	25.80	-	12.42	8.40	3.94
(*S*)-BPA 30%	25.34	-	12.53	8.40	3.93
(*S*)-BPA 50%	25.34	-	12.58	8.33	3.95

b

(Å)	In-plane (IP)					
	(100)	*(001)*	*(200)*	*(300)*	*(002)*	*(004)*
BrB	25.08	13.71	12.61	8.26	7.00	3.52
(*S*)-BPA 10%	25.31	13.84	-	-	7.03	3.53
(*S*)-BPA 30%	25.31	13.84	-	-	7.02	3.54
(*S*)-BPA 50%	25.08	13.91	-	-	7.09	3.56

c

(nm)	Crystalline coherence length (CCL)			
	IP *(100)*	IP *(001)*	OOP *(100)*	OOP *(010)*
BrB	20.49	11.92	-	7.05
(*S*)-BPA 10%	14.82	26.98	6.94	2.92
(*S*)-BPA 30%	15.23	48.43	14.75	2.73
(*S*)-BPA 50%	16.41	78.06	15.74	2.99

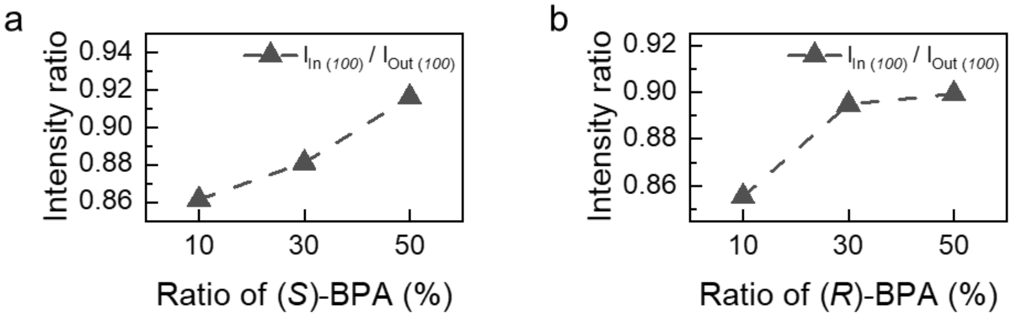

Figure S14. In-plane to out-of-plane (*100*) peak intensity ratio of a) (*S*)-BPA ratio, and b) (*R*)-BPA ratio.

Table S5. In-plane to out-of-plane CCL ratio via BPA ratio.

	In-plane to out-of-plane CCL ratio					
	(*S*)-BPA			(*R*)-BPA		
BPA ratio (%)	10%	30%	50%	10%	30%	50%
(*100*)	2.14	1.03	1.04	2.21	1.19	1.40

Table S6. Herman's orientation parameter via BPA ratio.

	Herman's Orientation Parameter (HOP)						
	BrB	(*S*)-BPA			(*R*)-BPA		
BPA ratio (%)	-	10%	30%	50%	10%	30%	50%
(*100*)	-0.33	0.10	0.10	0.05	0.10	0.08	0.07
(*010*)	0.26	0.06	0.06	0.05	0.09	0.08	0.09

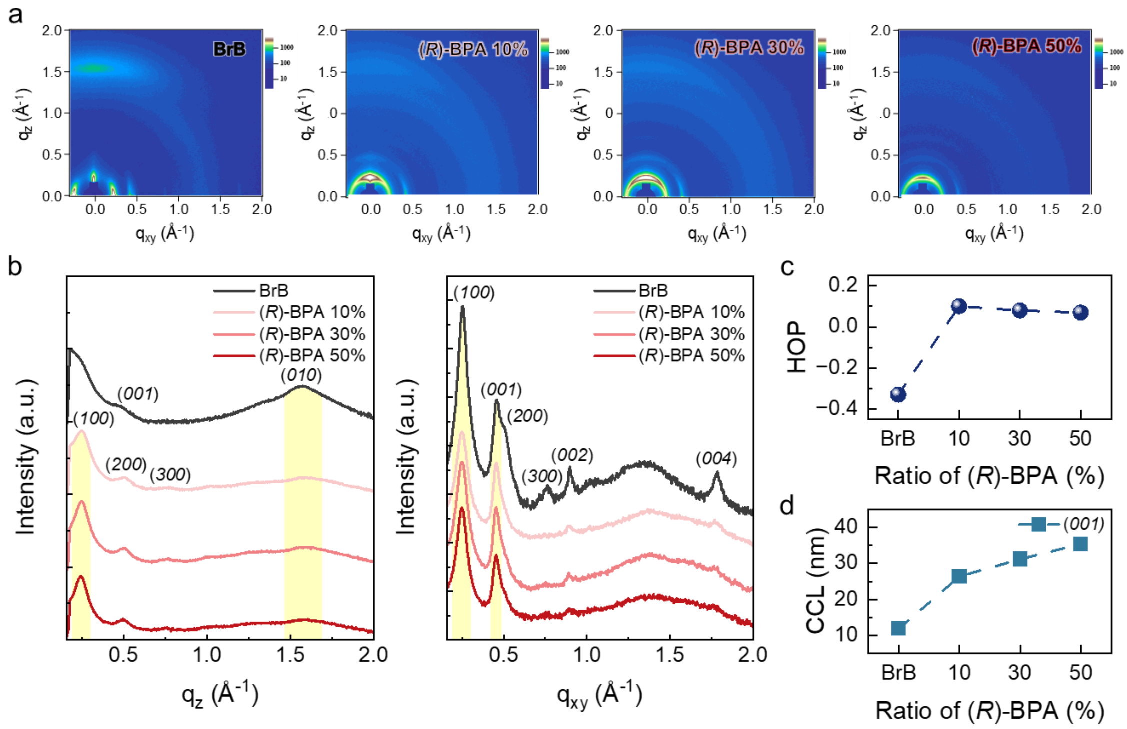

Figure S15. a) 2D-GIWAXS profile images depending on (*R*)-BPA ratio. b) Linecut graph with P(NDI2OD-T2) peaks assigned (the yellow shaded areas indicate FWHM for CCL calculation). c) Herman's orientation parameters of (*100*) d) CCL in in-plane at the (*001*) crystal peaks.

Table S7. d-spacing peaks for a) out-of-plane, b) in-plane, and c) crystalline coherence length as a function of (*R*)-BPA ratio.

a

(Å)	Out-of-plane (OOP)				
	(100)	*(001)*	*(200)*	*(300)*	*(010)*
BrB	-	13.93	-	-	4.00
(*R*)-BPA 10%	25.56	-	12.31	8.19	3.94
(*R*)-BPA 30%	25.56	-	12.42	8.14	3.94
(*R*)-BPA 50%	26.03	-	12.75	8.40	3.96

b

(Å)	In-plane (IP)					
	(100)	*(001)*	*(200)*	*(300)*	*(002)*	*(004)*
BrB	25.08	13.71	12.61	8.26	7.00	3.52
(*R*)-BPA 10%	25.31	13.84	-	-	7.07	3.55
(*R*)-BPA 30%	25.31	13.84	-	-	7.07	3.56
(*R*)-BPA 50%	25.31	13.91	-	-	7.00	3.57

c

(nm)	CCL, Parallel			
	IP *(100)*	IP *(001)*	OOP *(100)*	OOP *(010)*
BrB	20.49	11.92	-	7.05
(*R*)-BPA 10%	17.98	26.37	8.12	5.51
(*R*)-BPA 30%	18.37	31.21	15.43	2.93
(*R*)-BPA 50%	19.77	35.37	14.15	6.03

Section 7. Magneto field-effect transistor

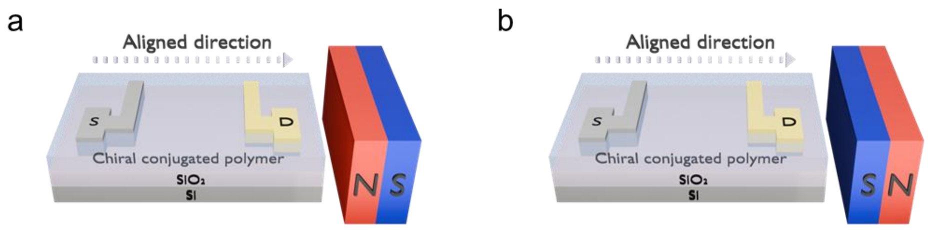

Figure S16. Schematic of mFET structures and measurement for a) N pole, and b) S pole.

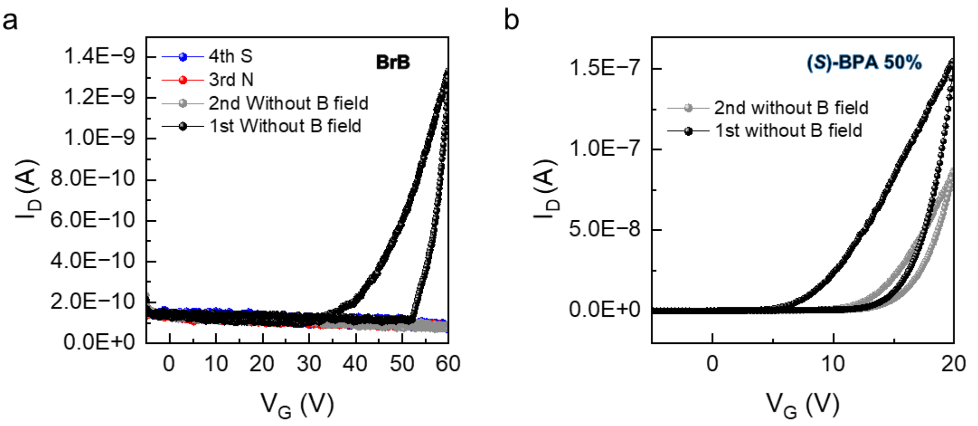

Figure S17. Transfer curves from multiple measurements for a) BrB, and b) (*S*)-BPA 50%.

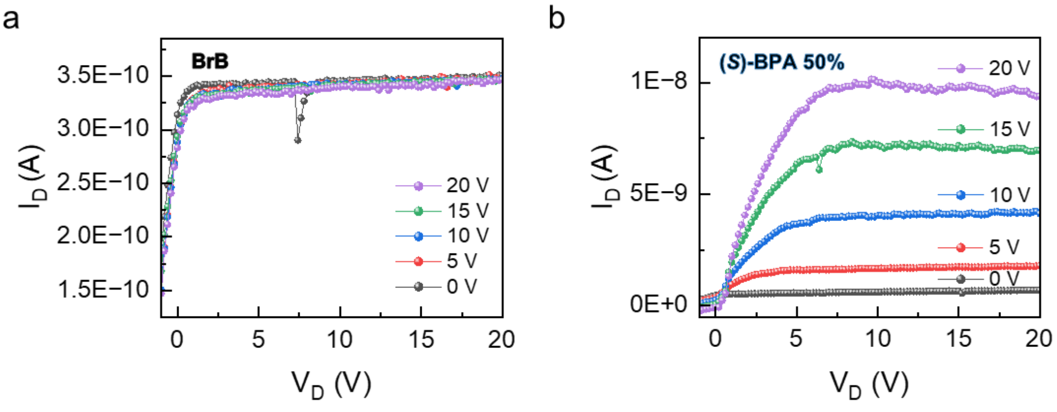

Figure S18. Output curve for a) BrB, and b) (*S*)-BPA 50% as a function of gate voltage.

Table S8. Field-effect transistor performance of BrB, (*S*)-BPA 50%, and (*R*)-BPA 50% with and without magnetic field.

* Number of measurements > 20

	μ_{avg} ($cm^2V^{-1}s^{-1}$)			V_{th} (V)		
	Without B field	N	S	Without B field	N	S
BrB	6.8 × 10^{-7} (± 1.8 × 10^{-7})	-	-	30.4	-	-
(*S*)-BPA 50%	1.7 × 10^{-4} (± 2.0 × 10^{-4})	2.1 × 10^{-4} (± 2.3 × 10^{-4})	2.0 × 10^{-4} (± 2.4 × 10^{-4})	10.0	7.9	8.1
(*R*)-BPA 50%	4.8. × 10^{-5} (± 4.0 × 10^{-5})	8.1 × 10^{-5} (± 2.5 × 10^{-5})	8.6 × 10^{-5} (± 2.6 × 10^{-5})	18.5	12.1	12.0

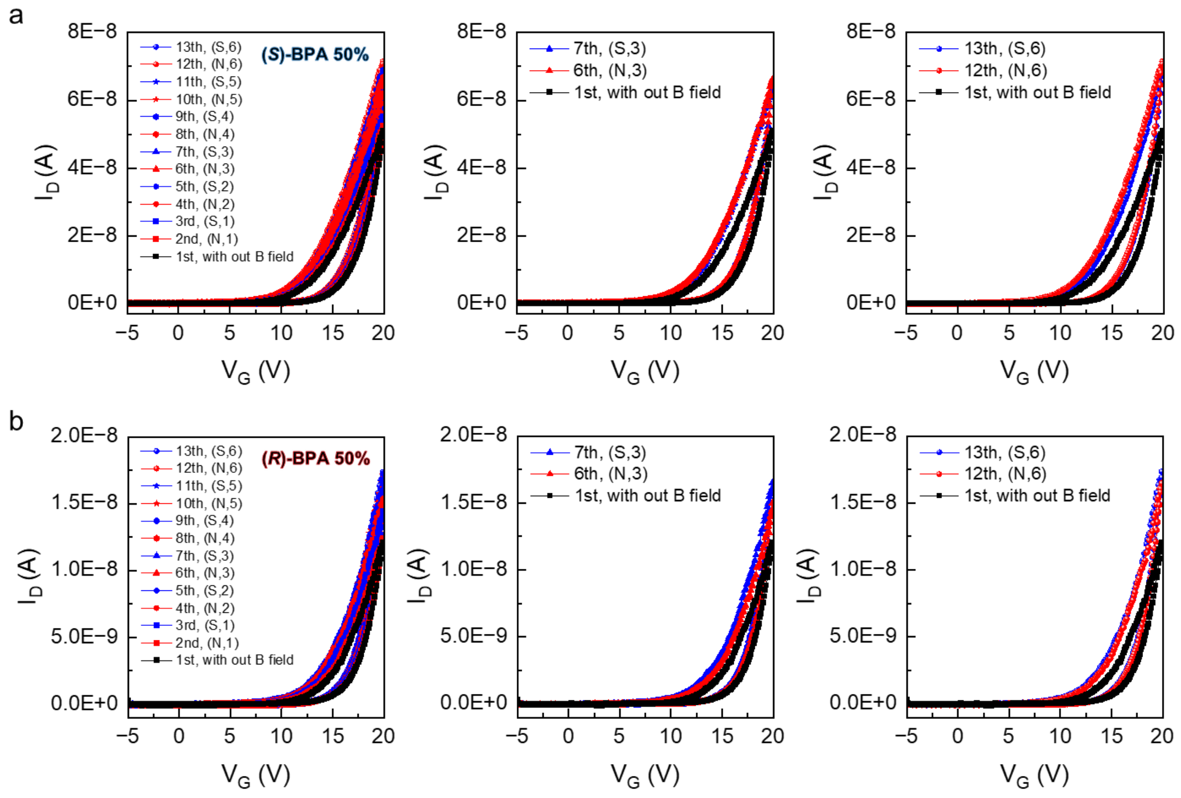

Figure S19. Transfer curves from consecutive measurements under north and south magnetic fields, and selected curves for a) (*S*)-BPA 50%, and b) (*R*)-BPA 50%.

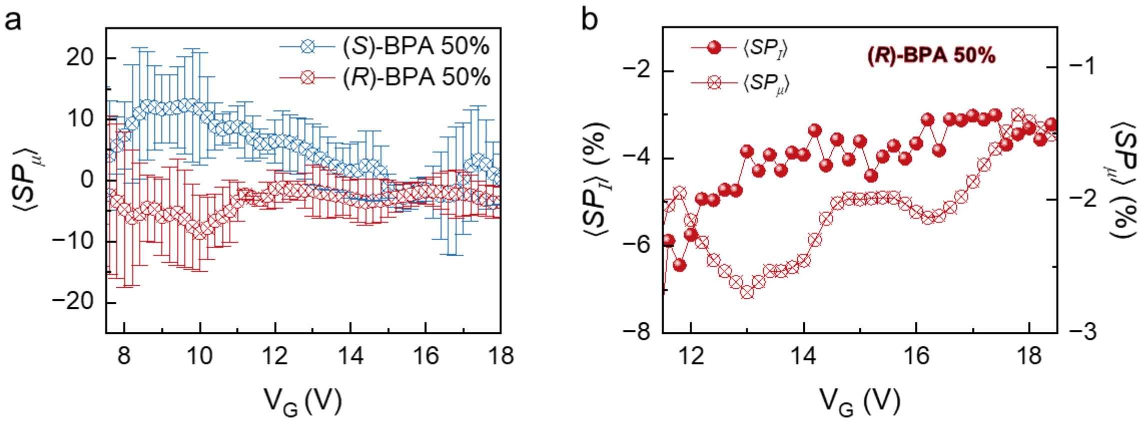

Figure S20. a) Spin polarization mobility with standard deviation of (*S*)-BPA 50%, and (*R*)-BPA 50%. b) Average spin polarization current and mobility of (*R*)-BPA 50%.

(1) Wang, S.; Li, H.; Zhao, K.; Zhang, L.; Zhang, Q.; Yu, X.; Tian, H.; Han, Y. Increasing the Charge Transport of P(NDI2OD-T2) by Improving the Polarization of the NDI2OD Unit along the Backbone Direction and Preaggregation via H-Bonding. Macromolecules 2022, 55 (7), 2497–2508. https://doi.org/10.1021/acs.macromol.1c02329.
(2) Giussani, E.; Fazzi, D.; Brambilla, L.; Caironi, M.; Castiglioni, C. Molecular Level Investigation of the Film Structure of a High Electron Mobility Copolymer via Vibrational Spectroscopy. Macromolecules 2013, 46 (7), 2658–2670. https://doi.org/10.1021/ma302664s.
(3) Smilgies, D.-M. Scherrer Grain-Size Analysis Adapted to Grazing-Incidence Scattering with Area Detectors. J. Appl. Crystallogr. 2009, 42 (6), 1030–1034. https://doi.org/10.1107/S0021889809040126.
(4) Tang, L.; McNeill, C. R. Capturing the Phase Transformation and Thermal Behavior of P(NDI2OD-T2) with In Situ Grazing Incidence WAXS. Macromolecules 2022, 55 (16), 7273–7283. https://doi.org/10.1021/acs.macromol.2c01025.
